# Supplementary material for: Epistatic Net allows the sparse spectral regularization of deep neural networks for inferring fitness functions
Source: Nat Commun. 2021 Sep 1;12:5225. doi: 10.1038/s41467-021-25371-3 (PMC8410946; doi:10.1038/s41467-021-25371-3)
Supplement: Supplementary file 1 — Supplementary Information [file 41467_2021_25371_MOESM1_ESM.pdf]

Supplementary Information

Epistatic Net Allows the Sparse Spectral Regularization of Deep Neural Networks for Inferring Fitness Functions

Amirali Aghazadeh[1], Hunter Nisonoff[2], Orhan Ocal[1], David H. Brookes[3], Yijie Huang[1], O. Ozan Koyluoglu[1], Jennifer Listgarten[1,2], and Kannan Ramchandran[1,*]

[1]Department of Electrical Engineering and Computer Science,
[2]Center for Computational Biology, [3]Biophysics Graduate Group,
University of California, Berkeley
*Correspondence to: Kannan Ramchandran: kannanr@eecs.berkeley.edu

1 Supplementary Notes

1.1 Sparse recovery using sparse-graph codes

The problem we are interested in this section is recovering the WH transform (WHT) coefficients (equivalently, the pseudo-Boolean function) when there is sparsity in the WHT domain. Methods proposed in compressed sensing literature can be used to recover a sparse signal (i.e., landscape) in a sample efficient way [1]. However, the algorithms proposed in the literature like Orthogonal Matching Pursuit (OMP) [2] or Lasso [3] requires operations that scale at least linearly with the ambient dimension p. On the other hand, our method requires sublinear computational complexity whenever the degrees of freedom k scales sub-linearly with the ambient dimension p [4]. The key properties of our algorithm are presented in the following theorem.

Theorem 1.1 ([4]). *Let $\alpha \in (0, 1)$ be a fixed number. Suppose $p = 2^d$ and assume $k = p^\alpha$. Let $\mathbf{y} \in \mathbb{R}^p$ be a vector and $\mathbf{Y} \in \mathbb{R}^p$ be its WHT. Assume that $\mathbf{Y}$ is k-sparse and its support is selected uniformly at random among all possible $\binom{d}{k}$ subsets of $[d]$ of size k. Then, there is an algorithm with the following properties:*

1. *Sample complexity: Algorithm uses $\mathcal{O}(k \log^2 p)$ samples of $\mathbf{y}$.*
2. *Computational complexity: Total number of operations to successfully decode all nonzero WHT coefficients or declare a decoding failure is $\mathcal{O}(k \log^3 p)$.*
3. *Success probability: Probability of recovering $\mathbf{Y}$ completely approaches 1 as p grows, where the probability is taken over randomness of selecting the support of $\mathbf{Y}$.*

This speedup is achieved by employing a divide-and-conquer strategy where we break the problem of recovering a k-sparse signal into k smaller problems of recovering 1-sparse signal, solve each 1-sparse problem efficiently, and then combine the solutions to each of them to recover the original signal. The recovery algorithm is closely tied to decoding a sparse-graph code through peeling using techniques from the literature on Low Density Parity Check (LDPC) codes [5] and product codes [6].

Note that under the assumptions of the theorem, theoretically, order of $k \log(p)$ samples are required for learning the correct model by information theoretic arguments [4]. The algorithm described here, which requires $k \log^2(p)$ samples is off from order optimality by only a logarithmic factor. As a matter of fact, the algorithm can be tweaked to be order optimal [4]. However, that version of the algorithm is not described in this paper as it requires a complex additional step.

The first step of the algorithm is to generate linear mixing of transform domain coefficients based on the following property.

Property 1. *Let* $\mathbf{y}$ *be a* $p = 2^d$ *length vector. Given a shift vector* $\mathbf{q} \in \mathbb{F}_2^d$ *and a full-rank subsampling matrix* $\mathbf{H} \in \mathbb{F}_2^{b\times d}$*, let* $\mathbf{z}$ *be the vector of length* $B = 2^b$ *where* $\mathbf{z}_{\mathbf{x}} = \mathbf{y}_{\mathbf{x}\mathbf{H}+\mathbf{q}}$ *for all* $\mathbf{x} \in \mathbb{F}_2^b$*. Then, the WHT coefficients of* $\mathbf{z}$ *satisfy*

$$\mathbf{Z}_{\mathbf{k}} = \sqrt{\frac{B}{p}} \sum_{\mathbf{j}\in\mathbb{F}_2^p:\mathbf{j}\mathbf{H}^\top=\mathbf{k}} (-1)^{\langle \mathbf{q},\mathbf{j}\rangle} \mathbf{Y}_{\mathbf{j}}, \tag{1}$$

where $\mathbf{Y}_{\mathbf{j}}$ *is the* $\mathbf{j}^{th}$ *WHT coefficient of* $\mathbf{y}$.

The above property states that the WHT coefficients $\mathbf{Y}_{\mathbf{k}}$ are modulated by $(-1)^{\langle \mathbf{q},\mathbf{k}\rangle}$ when a shift of $\mathbf{q}$ is applied to the indices of $\mathbf{y}$, and that subsampling of the input signal creates a linear mixing of WHT coefficients.

Using Property 1 we create linear mixing of coefficients by choosing C many subsampling matrices $\mathbf{H}_1, \cdots, \mathbf{H}_C$ where each matrix is $b \times d$ dimensional. Furthermore, we choose for each subsampling $\mathbf{P}_1, \cdots, \mathbf{P}_C$ shift matrices where each of them is $\mathcal{O}(\log^2 p) \times d$ dimensional. The choice of C, the matrices $\mathbf{H}_i$ and the delays $\mathbf{P}_i$ for $i = 1, \cdots, C$ are going to be described in the following sections. Then WHT coefficients are calculated for the shifted-and-subsampled sequences. We give an example below for the linear mixing resulting from subsampling.

Example 1. *Let* $\mathbf{y}$ *be a vector of length* 16*, and let us define* $\mathbf{z}_{\mathbf{x}}^{(1)} = 2\mathbf{y}_{\mathbf{H}_1\mathbf{x}}$ *and* $\mathbf{z}_{\mathbf{x}}^{(2)} = 2\mathbf{y}_{\mathbf{H}_2\mathbf{x}}$ *where*

$$\mathbf{H}_1 = \begin{pmatrix} 0 & 0 \\ 0 & 0 \\ 1 & 0 \\ 0 & 1 \end{pmatrix}, \mathbf{H}_2 = \begin{pmatrix} 1 & 0 \\ 0 & 1 \\ 0 & 0 \\ 0 & 0 \end{pmatrix}.$$

From property 1, we see that all the WHT coefficients of $\mathbf{y}$ *whose binary index have the same last two digits is hashed to the same bin (underlined in the following equations) for* $\mathbf{z}^{(1)}$*, that is, we have*

$$\begin{aligned}
\mathbf{Z}_{00}^{(1)} &= \mathbf{Y}_{00\underline{00}} + \mathbf{Y}_{01\underline{00}} + \mathbf{Y}_{10\underline{00}} + \mathbf{Y}_{11\underline{00}}, \\
\mathbf{Z}_{01}^{(1)} &= \mathbf{Y}_{00\underline{01}} + \mathbf{Y}_{01\underline{01}} + \mathbf{Y}_{10\underline{01}} + \mathbf{Y}_{11\underline{01}}, \\
\mathbf{Z}_{10}^{(1)} &= \mathbf{Y}_{00\underline{10}} + \mathbf{Y}_{01\underline{10}} + \mathbf{Y}_{10\underline{10}} + \mathbf{Y}_{11\underline{10}}, \\
\mathbf{Z}_{11}^{(1)} &= \mathbf{Y}_{00\underline{11}} + \mathbf{Y}_{01\underline{11}} + \mathbf{Y}_{10\underline{11}} + \mathbf{Y}_{11\underline{11}}.
\end{aligned}$$

Similarly, for $\mathbf{z}^{(2)}$ *we get*

$$
\begin{aligned}
\mathbf{Z}^{(2)}_{00} &= \mathbf{Y}_{\underline{00}00} + \mathbf{Y}_{\underline{00}01} + \mathbf{Y}_{\underline{00}10} + \mathbf{Y}_{\underline{00}11}, \\
\mathbf{Z}^{(2)}_{01} &= \mathbf{Y}_{\underline{01}00} + \mathbf{Y}_{\underline{01}01} + \mathbf{Y}_{\underline{01}10} + \mathbf{Y}_{\underline{01}11}, \\
\mathbf{Z}^{(2)}_{10} &= \mathbf{Y}_{\underline{10}00} + \mathbf{Y}_{\underline{10}01} + \mathbf{Y}_{\underline{10}10} + \mathbf{Y}_{\underline{10}11}, \\
\mathbf{Z}^{(2)}_{11} &= \mathbf{Y}_{\underline{11}00} + \mathbf{Y}_{\underline{11}01} + \mathbf{Y}_{\underline{11}10} + \mathbf{Y}_{\underline{11}11}.
\end{aligned}
$$

Under the assumptions of Theorem 1.1 on sparsity and the support of the non-zero WHT coefficients of the signal, the linear mixing of coefficients take a form where they can be solved for through peeling. The following provides an example of such linear mixing.

Example 2. *Let* $\mathbf{y} \in \mathbb{R}^{16}$ *have WHT coefficients equal to*

$$
\mathbf{Y}_{\mathbf{k}} = \begin{cases} \mathbf{Y}_{0001} & \text{if } \mathbf{k} = 0001, \\ \mathbf{Y}_{0100} & \text{if } \mathbf{k} = 0100, \\ \mathbf{Y}_{0101} & \text{if } \mathbf{k} = 0101, \\ \mathbf{Y}_{1010} & \text{if } \mathbf{k} = 1010, \\ 0 & \text{otherwise.} \end{cases}
$$

Under the subsampling used in example 1 the WHT coefficients of the sub-sampled vectors satisfy

$$
\begin{aligned}
\mathbf{Z}^{(1)}_{00} &= \mathbf{Y}_{01\underline{00}}, & \mathbf{Z}^{(2)}_{00} &= \mathbf{Y}_{\underline{00}01}, \\
\mathbf{Z}^{(1)}_{01} &= \mathbf{Y}_{00\underline{01}} + \mathbf{Y}_{01\underline{01}}, & \mathbf{Z}^{(2)}_{01} &= \mathbf{Y}_{\underline{01}00} + \mathbf{Y}_{\underline{01}01}, \\
\mathbf{Z}^{(1)}_{10} &= \mathbf{Y}_{10\underline{10}}, & \mathbf{Z}^{(2)}_{10} &= \mathbf{Y}_{\underline{10}10}, \\
\mathbf{Z}^{(1)}_{11} &= 0, & \mathbf{Z}^{(2)}_{11} &= 0.
\end{aligned}
$$

We give the details of peeling algorithm in reference to this example in the following section.

1.2 Recovery Through Peeling with an Oracle

The relationship between the measurements and the unknown coefficients can be shown as a bipartite graph. The graph related to the linear mixing in Example 2 and the recovery of the non-zero coefficients are illustrated in Figure S1. The unknown coefficients are shown on the left and referred to as variable nodes, and the measurements are shown on the right and referred to as check nodes. An edge is drawn

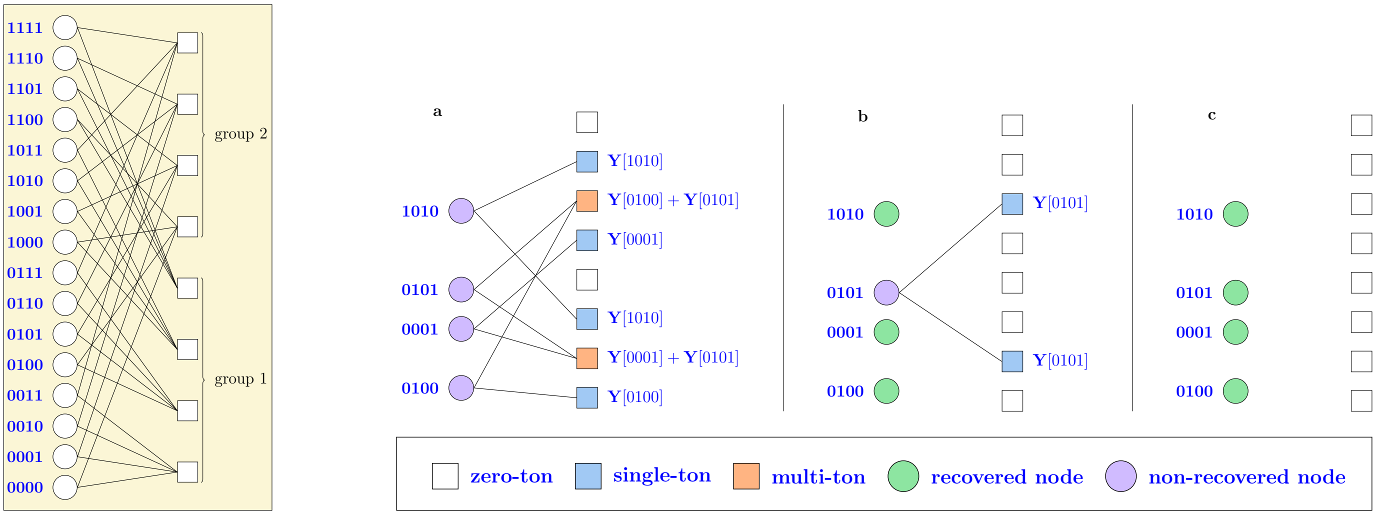

Figure S1: **(Left)** The connections between the variable nodes (WHT coefficients) and the check nodes (measurements) in Example 1. **(Right)** Recovering the unknown coefficients in Example 2. The graph induced by the non-zero coefficients is shown in **a**. In the first round of peeling we recover coefficients at indices 0100, 001 and 1010, and get the graph in **b**. In two rounds of peeling, all the non-zero elements of the signal are recovered as shown in **c**.

between a variable node and a check node if the unknown coefficient related to that variable node contributes to the measurement related to that check node. Each check node can be categorized into the following three types:

1. Zero-ton: a check node is a zero-ton if it has no non-zero coefficients (shaded in white in Figure S1).
2. Single-ton: a check node is a single-ton if it involves only one non-zero coefficient (shaded in blue in Figure S1). Specifically, we refer to the index k and its associated value $\mathbf{Y_k}$ as the index-value pair $(\mathbf{k}, \mathbf{Y_k})$.
3. Multi-ton: a check node is a multi-ton if it contains more than one non-zero coefficient (shaded in orange in Figure S1).

To illustrate the peeling algorithm for recovery, we assume that there exists an "oracle" that informs the decoder exactly which check nodes are single-tons, and provides the index-value pair for that single-ton. In Example 2, in the first round of peeling (shown in Panel (A) in Figure S1), the oracle informs the decoder that the check nodes corresponding to $\mathbf{Z}_{00}^{(1)}$, $\mathbf{Z}_{10}^{(1)}$, $\mathbf{Z}_{00}^{(2)}$, and $\mathbf{Z}_{10}^{(2)}$ are single-tons with index-value pairs $(0100, \mathbf{Y}_{0100})$, $(1010, \mathbf{Y}_{1010})$, $(0001, \mathbf{Y}_{0001})$ and $(1010, \mathbf{Y}_{1010})$ respectively. Then the decoder can subtract their contributions from other check nodes, forming new single-tons. Therefore, with the oracle information, the peeling decoder repeats the following steps:

1. select all the edges in the bipartite graph with right degree 1 (identify single-ton bins);

2. remove (peel off) these edges as well as the corresponding pair of variable and check nodes connected to these edges;
3. remove (peel off) all other edges connected to the variable nodes that have been removed in Step 2.
4. subtract the contributions of the variable nodes from the check nodes whose edges have been removed in Step 3.

Decoding is successful if all the edges are removed from the graph.

In this work we choose the subsampling matrices uniformly at random over $\mathbb{F}^{b\times d}$. Other constructions alongside with their theoretical guarantees can be found at [4, 7]. We chose the random design as it is observed to have superior practical performance in some regimes of interest [7, 8].

Since the proof of the algorithm follows the same steps as in [4], we just provide a sketch here and refer the interested readers to that paper. Since the sparsity is uniformly distributed, each non-zero entry of $\mathbf{Y}$ is connected to a check node chosen uniformly at random in each subsampling group. This results in a left-regular LDPC code construction, and the proof for recovering the support $\mathbf{Y}$ follows the same steps in [4].

Table S1: Thresholds for recovery [4]. M : number of check nodes, k : number of variable nodes (sparsity).

groups	3	4	5	6
M/k	1.2218	1.2949	1.4250	1.5697

In peeling, we recover a variable node (non-zero coefficient of $\mathbf{Y}$) if it is connected to a check node with degree 1, and remove the outgoing edges from that variable node. The density evolution is a powerful tool in modern coding theory that tracks the average density of remaining edges in the graph after ℓ rounds of peeling [5]. The density evolution equations for our setting is given by the recursive equation

$$p_\ell = \left(1 - e^{-vp_{\ell-1}/(M/k)}\right)^{v-1}, \tag{2}$$

where $p_0 = 1$, and M is the total number of parity check nodes. This assumes that the depth ℓ neighborhood of the chosen edge is a tree. We can show similarly to [4] that the depth ℓ neighborhood of a randomly chosen edge is a tree with high probability for any fixed ℓ. On average, an arbitrarily large fraction of edges are removed if p_ℓ goes to zero as $\ell \to \infty$. For p_ℓ to go to zero, M/k needs to be greater than a threshold for a fixed v. These thresholds are shown in Table S1. Then, one can use the standard Doob's martingale argument to show that the fraction of non-recovered components concentrates around it's mean [9]. This guarantees recovery of arbitrarily-large fraction of significant components. Then, an expander-graph argument is used to show that peeling continues until all of the coefficients are recovered [4].

1.3 Replacing the oracle

We now show how to replace the oracle in the peeling algorithm with a realizable mechanism. This is done by employing $\mathcal{O}(\log^2 p)$ shifts for each subsampling matrix where $\log(p)$ shifts are to recover each digit of the location $\mathbf{k}$, and we take $\mathcal{O}(\log p)$ samples for each location for noise averaging. Let $\mathbf{U}_{\mathbf{H},\mathbf{q}}(\mathbf{k})$ be the $\mathbf{k}^{\text{th}}$ WHT coefficient of the signal obtained by shifting indices of $\mathbf{y}$ by $\mathbf{q}$ and then subsampling by $\mathbf{H}$. From Property 1 we have

$$\mathbf{U}_{\mathbf{H},\mathbf{q}}(\mathbf{k}) := \sqrt{\frac{B}{p}} \sum_{\mathbf{j}:\mathbf{j}\mathbf{H}^\top=\mathbf{k}} (-1)^{\langle \mathbf{j},\mathbf{q}\rangle} \mathbf{Y}_{\mathbf{j}}. \tag{3}$$

Furthermore, let us define the ratio of a WHT coefficient obtained by using the same subsampling matrix but using two different shifts

$$\mathbf{r}_{\mathbf{A},\mathbf{p},\mathbf{q}}(\mathbf{k}) := \frac{\mathbf{U}_{\mathbf{A},\mathbf{p}+\mathbf{q}}(\mathbf{k})}{\mathbf{U}_{\mathbf{A},\mathbf{p}}(\mathbf{k})}. \tag{4}$$

Assume that for a WHT index $\mathbf{k}$ in equation (3), there is only one index $\mathbf{j}$ such that $\mathbf{A}^\top\mathbf{j} = \mathbf{k}$ and $\mathbf{Y}_{\mathbf{j}} \neq 0$ (that is, the check node corresponding to it is a single-ton). Then, it follows that $\mathbf{U}_{\mathbf{A},\mathbf{p}}(\mathbf{k}) = \sqrt{\frac{B}{p}}(-1)^{\langle \mathbf{j},\mathbf{p}\rangle}\mathbf{Y}_j$. Using $\mathbf{q} = \mathbf{e}_i \in \mathbb{F}^d$ (the vector with all indices equal to 0 except for the ith index which is equal to 1) in equation (4) yields

$$\mathbf{r}_{\mathbf{A},\mathbf{p},\mathbf{e}_i}(\mathbf{k}) = \frac{(-1)^{\langle \mathbf{j},\mathbf{p}+\mathbf{e}_i\rangle}\mathbf{Y}_{\mathbf{j}}}{(-1)^{\langle \mathbf{j},\mathbf{p}\mathbf{Y}_{\mathbf{j}}\rangle}} = (-1)^{\langle \mathbf{j},\mathbf{e}_i\rangle}. \tag{5}$$

Note that this value is in $\{-1, +1\}$ for all p if there is no noise. As the value of $\langle \mathbf{j}, \mathbf{e}_i\rangle$ is equal to the ith index of the location $\mathbf{j} \in \mathbb{F}_2^d$, by using shifts $\{\mathbf{e}_i\}_{i=0}^{d-1}$ going through all indices of $\mathbf{j}$ we can recover it. When there is noise, it can be shown that by taking $\mathcal{O}(\log p)$ random shifts, the probability of detecting the location wrongly can be made polynomially small [4].

1.4 Related works

Fourier attribution priors. Sparse epistatic regularization in Epistatic Net (EN) is conceptually related to a recent work describing Fourier-transform-based attribution priors in deep neural networks (DNNs) [10]. It has been observed that, in the context of mapping DNA sequence to transcription factors (TF) binding and chromatin accessibility profiles, penalizing high-frequency components of the

Fourier spectrum, can improve the stability, interpretability, and performance of DNNs. The focus of this work is, however, on the regularization of DNN to promote sparsity in Fourier basis. In fact, our results show that other forms of regularization in the spectral domain (e.g., ℓ_2-norm instead of the ℓ_1-norm) are not beneficial for protein function prediction. Also distinct from these works, our regularization has a semisupervised flavor in imposing the ℓ_1-norm loss over the WH transform of the entire DNN landscape, that is, the combinatorial space of proteins which includes the ones that have not been observed in the training set.

Theoretical aspects of sparse WH recovery. From a theory perspective, our regularization scheme relates to sparse recovery algorithms and compressed sensing. One distinction is that the fitness function in proteins do not exactly follow the exact sparse signal model in compressed sensing with added Gaussian noise. Therefore, the classical compressed sensing bounds would not directly apply to the problem in practice. Approximate guarantees for sparse recovery would be an interesting theoretical direction especially in light of the improvements that we have observed over Lasso in terms of sample complexity with EN regularization. We speculate that DNN has an internal inductive bias in favor of natural fitness functions in biology that enable us to reduce the effective dimensioanlity of the problem and thus improve the sample complexity bounds over Lasso. More theoretical studies in this regard is deferred to future works.

Peeling algorithm. Our work also suggests a new method to generalize the use-case of recent peeling-decoding algorithms [11–14] for sparse-Fourier (WH) recovery problems to settings where we do not have the luxury to select (i.e., design) the sampling patterns based on codes. In such physically-constrained sampling scenarios, DNN can be trained on the data at hand and serve as a "jump-start" that interpolates the data so that it can be queried at any binary patterns. The SGD algorithm converges to a point in DNN that will induce some aliasing effect over the signal that would be interesting to be studied theoretically. Transfer learning has recently emerged as a powerful technique in training deep neural notworks in low-sample regime. In protein design, it has been shown that [15] one can use the wealth of unsuperivised protein data to find a new representation for proteins. Such representation enables training a neural network using handful of proteins for design purposes. In our paper we do consider any external unsupervised data. However it is an interesting question to investigate how much of the power gained in the new representation could have been explained by the sparsity assumption in WH basis.

1.5 Experiment on four canonical bacterial fitness

The fitness landscapes of *Escherichia coli*, *Aspergillus niger*, and β-lactam resistance [16–18] capture the effect of the absence/presence of $d = 5$ mutations which creates fitness landscapes of size $p = 2^{d=5} = 32$. We sampled $n = 10$ random data points from each landscape and used them to train the models. The fitness landscape of *Saccharomyces cerevisiae* growth [19] captures the effects of $d = 6$ mutations which creates a fitness landscapes of size $p = 2^{d=6} = 64$. We sampled $n = 20$ random data points from the landscape and used them to train the models.

1.6 Synthetic sparse fitness landscapes

We assessed the performance of our ℓ_1-norm WH (EN)-regularized DNN algorithm on three sets of easy, medium, and hard data sets, each comprising 12 synthetic fitness landscapes with $n = 13$ mutations. In terms of dimensions, we followed the real-world protein landscape of [20], however, we changed the order and type of interactions and their weightings. We considered sparse protein landscapes with $k = 8$ non-zero WH coefficients. We sampled the interactions randomly (with a uniform distribution) from a subset of WH coefficients with up to 2nd-order interactions for the "easy" data set. We selected one of the interactions randomly and replaced it by a random high-order interaction to make the "medium" data set and selected an additional three random interactions and replaced them by three random high-order interactions to make the "hard" data set. In all cases we set the weights of coefficient to be equal. We split the landscape (of size 8192) into training, validation, and test sets of sizes $n = 40$, 1000, and 1000, respectively. Fig. S2 shows the average accuracy of the algorithms in predicting fitness over 5 repeats of the experiments with random splits of the data into training/validation/test sets. Our ℓ_1-norm WH-regularized DNN consistently outperforms the DNN without WH regularization and the Lasso algorithm. The gap becomes even more distinct in presence of higher-order interactions.

2 Supplementary Figures

In this section we list and elaborate on the supplementary tables and figures used in the paper.

Table S2: Description of the biological landscapes used in this paper is tabulated in terms of genotype, phenotype, number of mutations, input sequence size (d), and the reference to the publication.

	**Genotype**	**Phenotype**	# sites (d)	**Reference**
D1	Scattered genomic mutations	*Escherichia coli* fitness	5 (5)	Khan et al. (2011) [16]
D2	Chromosomes in asexual fungi	*Aspergillus niger* fitness I	5 (5)	de Visser et al. (2009) [17]
D3	Protein point mutations	Resistance to β-lactam antibiotic	5 (5)	Weinreich et al. (2006) [18]
D4	Alleles in biosynthetic network	*Saccharomyces cerevisiae* haploid growth rate	6 (6)	Hall et al. (2010) [19]
D5	Protein mutations	*Entacmaea quadricolor* fluorescence	13 (13)	Poelwijk et al. (2019) [20]
D6	Protein mutations	I-binding domain of protein G (GB1)	4 (80)	Wu et al. (2014) [21]
D7	Protein mutations	*Aequorea victoria* green fluorescence	236 (236)	Sarkisyan et al. (2016) [22]

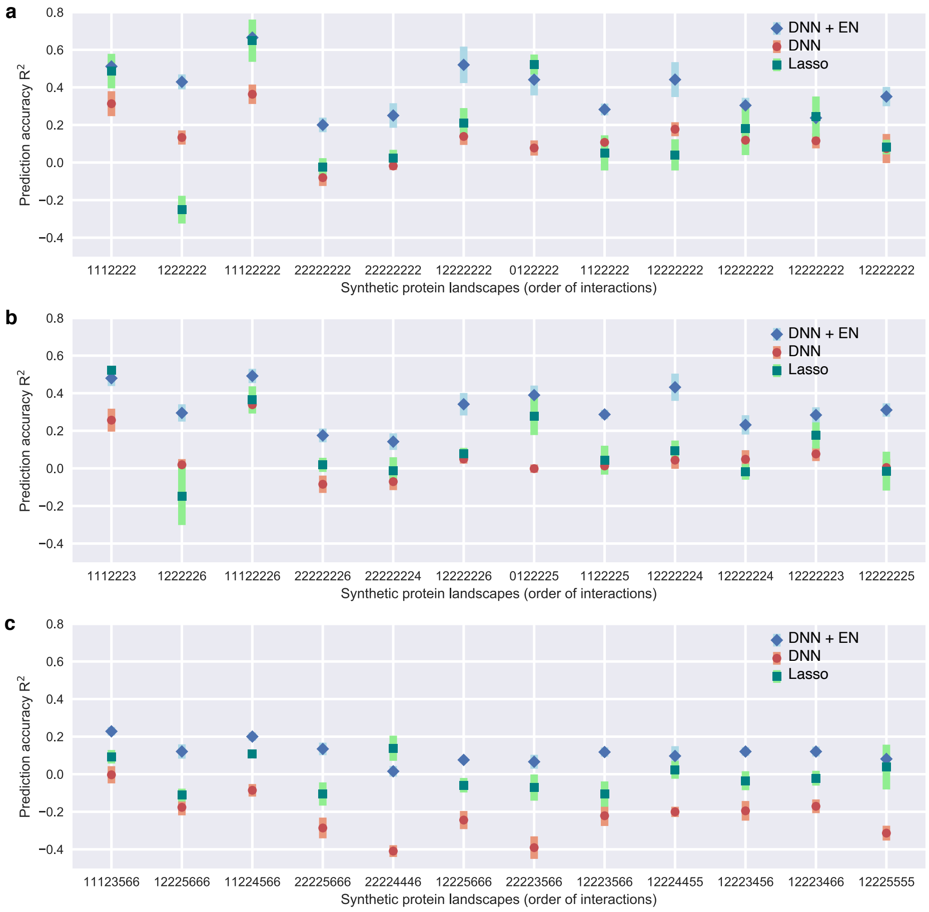

Figure S2: **Function prediction on synthetic landscapes with progressively more complex interactions. a**, Twelve landscapes with 8 interactions of up to order 2 are generated in WH basis and split randomly into training, validation, and test sets. Figure shows the prediction accuracy over the test set in 8 landscapes. Deep neural network (DNN) with Epistatic Net (EN) regularization outperforms DNN without regularization and Lasso regression. **b**, One of the interactions is selected from the landscapes in panel **a** at random and replaced with a high-order interactions. The experiments are repeated over the new more rugged landscapes. The prediction accuracy drops in all the algorithms due to ruggedness, however, DNN with EN regularization outperforms the competing baselines. **c**, An additional three interactions are selected at random and replaced with high-order interactions. DNN with WH-regularization has a consistently better or comparable prediction performance compared to DNN with no WH regularization and the Lasso algorithm in WH basis. All the experiments are repeated 5 times with random splits of the data into training, validation, and test sets. The error bars show the standard error of the mean (SEM).

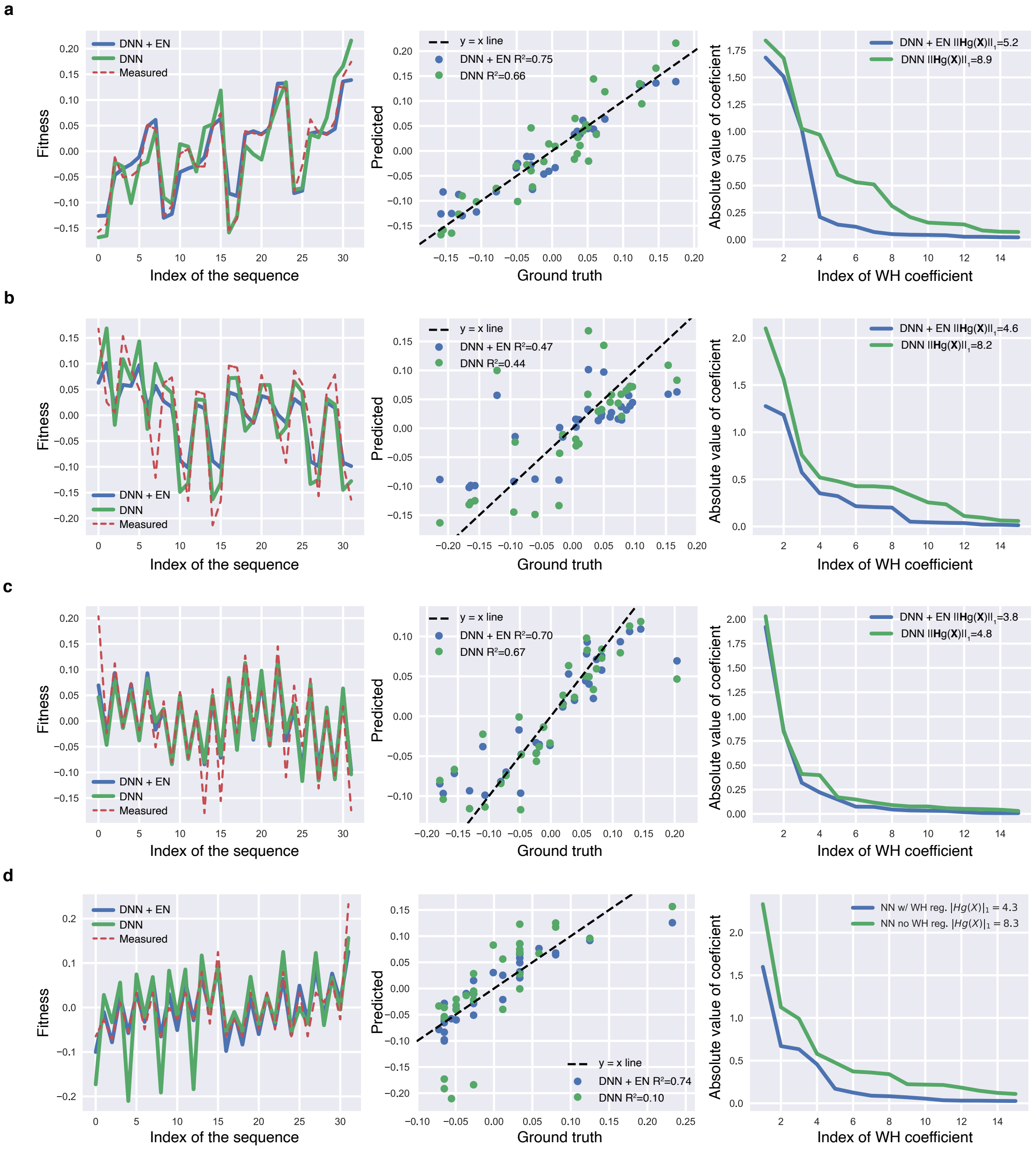

Figure S3: **Epistatic regularization in bacterial functions.** Analyzing the effect of the EN regularization on the WH transform and prediction accuracy of DNN trained on the fitness landscapes of **a**, Khan et al. [16], **b** and **c**, de Visser et al. [17, 23], **d**, Weinreich et al. [18] with $d = 5$ mutational sites. First and second columns compare the full landscapes predicted by DNN with and without EN to the experimentally measured ones. Third column compares the magnitude and ℓ_1-norm of the recovered epistasis. Models are trained on 10 random subset and tested on another 10 unseen random subset of the dataset. DNN with EN regularization has a consistently higher recovery accuracy of landscapes and lower ℓ_1-norm of recovered epistasis.

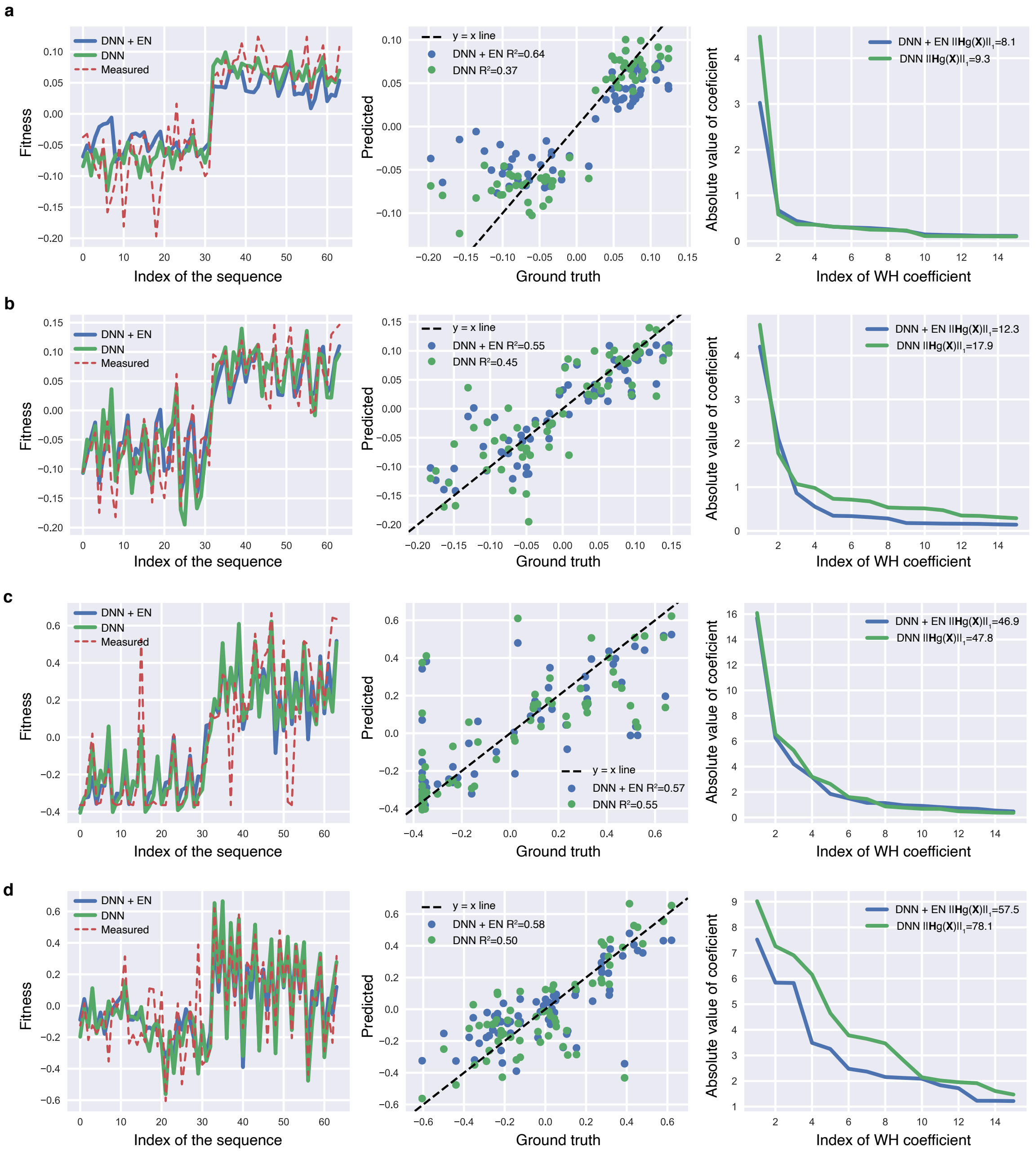

Figure S4: **Epistatic regularization in bacterial functions.** Analyzing the effect of EN regularization on the WH transform and prediction accuracy of DNN trained on the fitness landscapes of Hall et al. [19] with $d = 6$ mutational sites: **a**, Haploid growth, **b**, Diploid growth, **c**, Mating efficiency, and **d**, Sporulation. First and second columns compare the full landscapes predicted by DNN with and without EN to the experimentally measured ones. Third column compares the magnitude and ℓ_1-norm of the recovered epistasis. Models are trained on 20 random subset and tested on another 20 unseen random subset of the dataset. DNN with EN regularization has a consistently higher recovery accuracy of landscapes and lower ℓ_1-norm of recovered epistasis.

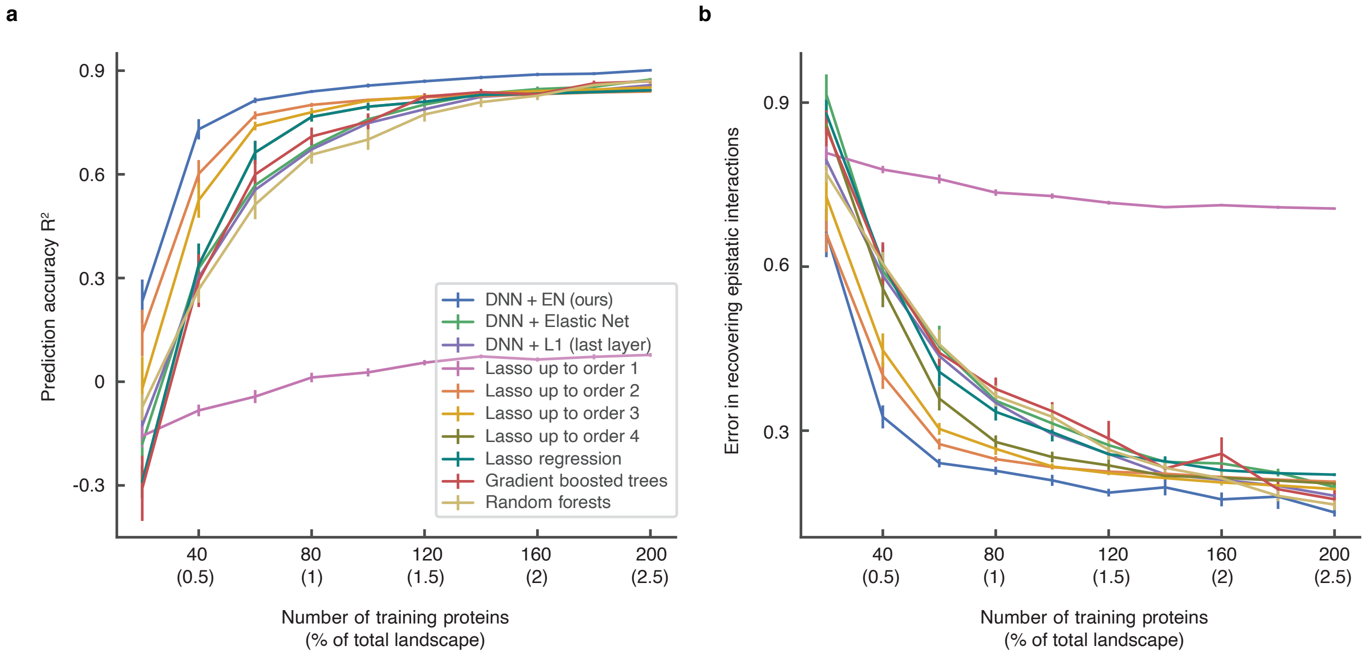

Figure S5: **Comparison of the prediction performance and epistatic recovery of DNN with the Epistatic Net (EN) regularization and DNNs with other forms of sparsity promoting regularizes in the *Entacmaea quadricolor* fluorescence landscape, over a wider range training set sizes.** **a**, The prediction accuracy of DNN with EN regularization is compared with the accuracy of DNNs with other forms of regularizations including ℓ_1 and ℓ_2-norm on the weights of DNN directly (Elastic Net regularization), and ℓ_1-norm on the last layer of DNN. **b**, The epistatic recovery performance of DNN with EN regularization is compared to the same competing algorithms. Both plots demonstrates that DNN with EN regularization maintains a consistent performance gap compared to all the baseline algorithms for a wide range of training sizes. Error bars show the standard error of the mean (SEM) in 20 independent repeats of the experiments with random splits of the data into training, validation, and test sets.

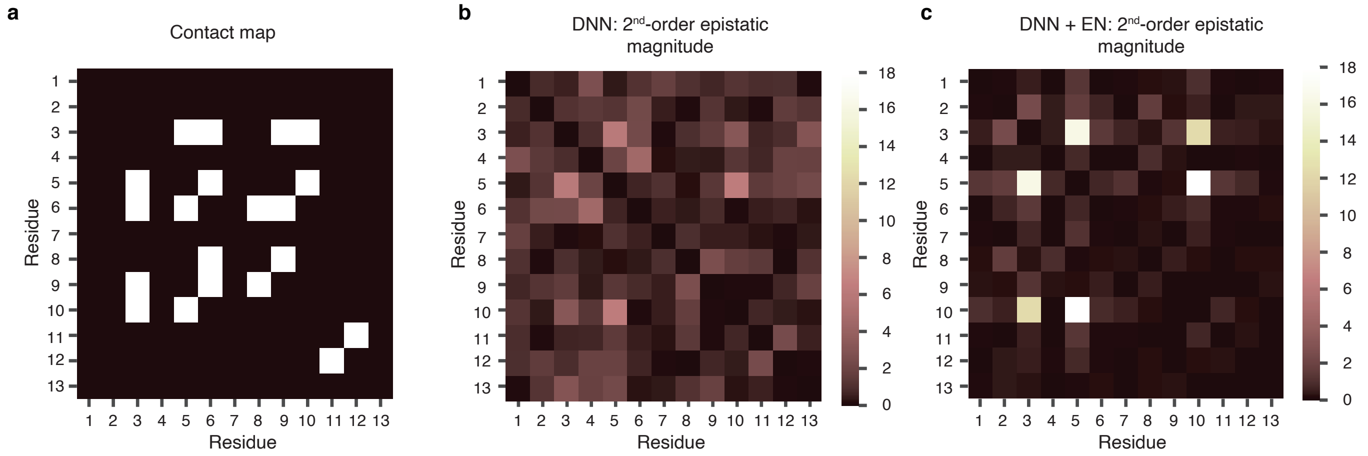

d

F1 score	DNN	DNN + EN
2nd order contact	0.68	0.76
3rd order contact	0.66	0.67

Figure S6: **Comparison of the DNN with and without the Epistatic Net (EN) regularization in recovering the 3D structure of the *Entacmaea quadricolor* fluorescence protein. a**, The contact map of the protein with residues within 4.5Å neighborhood of each other is visualized [24]. **b**, The second-order epistatic interactions recovered from the DNN trained on $n = 60$ labeled proteins shows several false interactions compared to the contact map ($F_1 = 0.68$). **c**, The second-order epistatic interactions recovered from the EN-regularized variant of DNN limits the false positive rate and improves the precision and recall rates ($F_1 = 0.76$). **d**, The third-order epistatic interactions of DNN with EN regularization predicts groups of three resides in contact (i.e., with smaller than 4.5Å pairwise distances) with higher F_1 score as well.

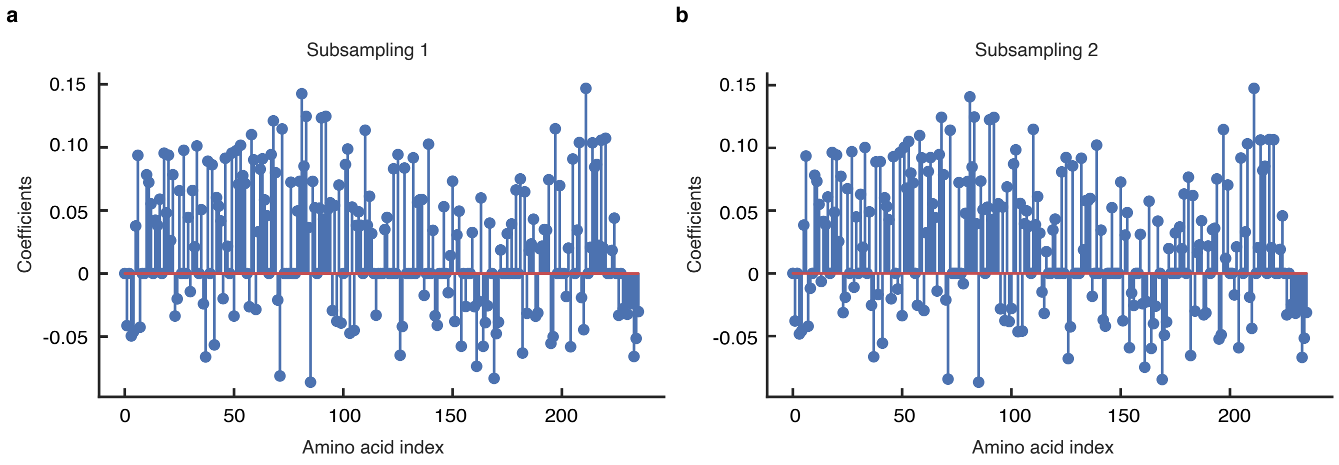

Figure S7: **Finding the epistatic interactions of deep neural networks (DNNs) using our Epistatic Net (EN) method under two independent subsamplings.** The coefficients of first-order interactions of DNN trained on the avGFP protein landscape recovered by EN-S is plotted under two independent subsamplings: subsampling pattern 1 in panel **a** and subsampling pattern 2 in panel **b**. The recovered coefficients are highly correlated ($R^2 = 0.99$) despite the enormous level of undersampling, that is, $5,074,944$ out of a total of 10^{71} sequences.

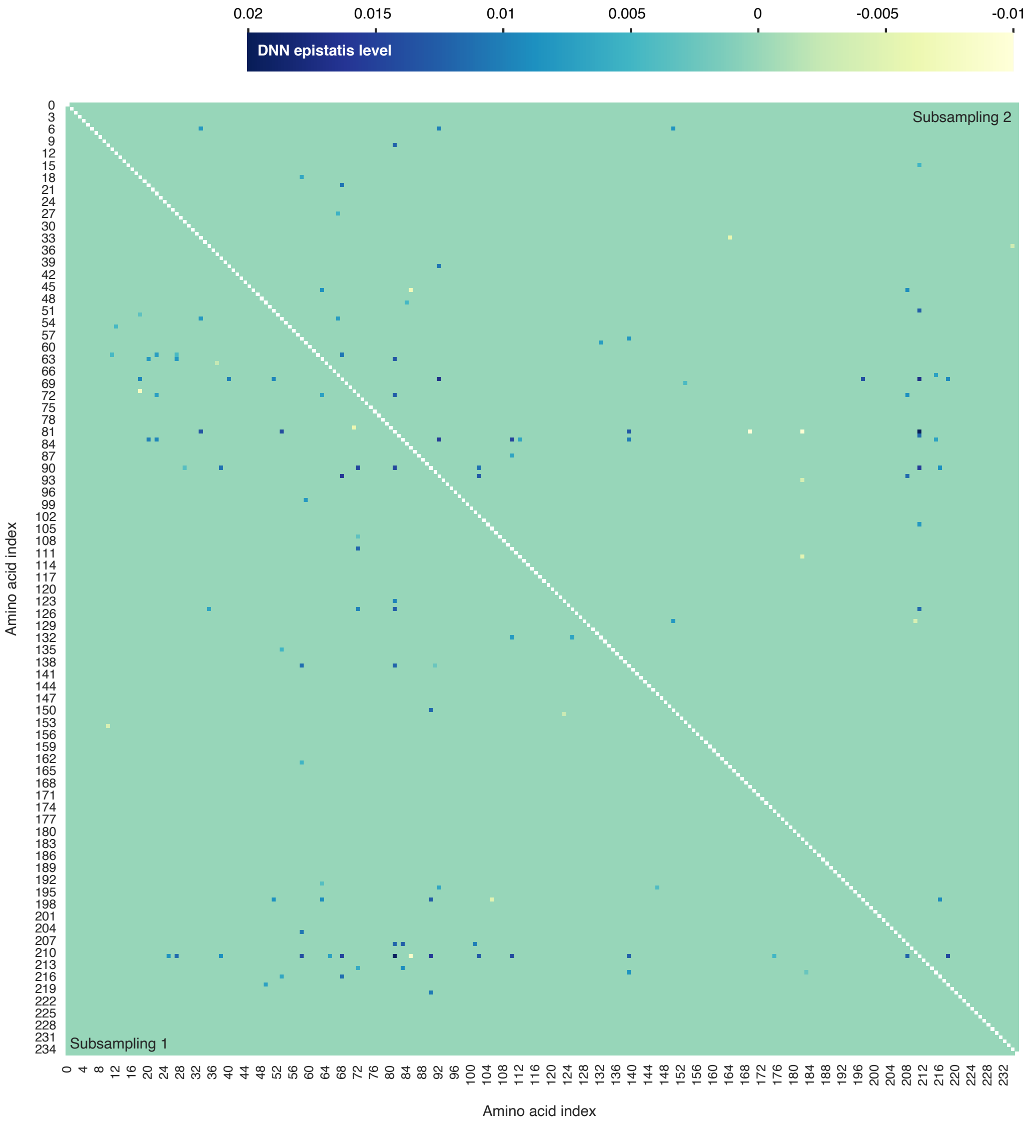

Figure S8: **Finding the epistatic interactions of deep neural networks (DNNs) using our Epistatic Net (EN) method under two independent subsamplings.** The coefficients of second-order interactions of DNN trained on the avGFP protein landscape recovered by EN-S is plotted under two independent subsamplings. The recovered coefficients are (locally-)correlated ($R^2 = 0.60$) despite the enormous level of undersampling, that is, $5,074,944$ out of a total of 10^{71} sequences. Local block-correlation is found by evaluating the correlation between 3×3 sub-blocks of the interaction matrices.

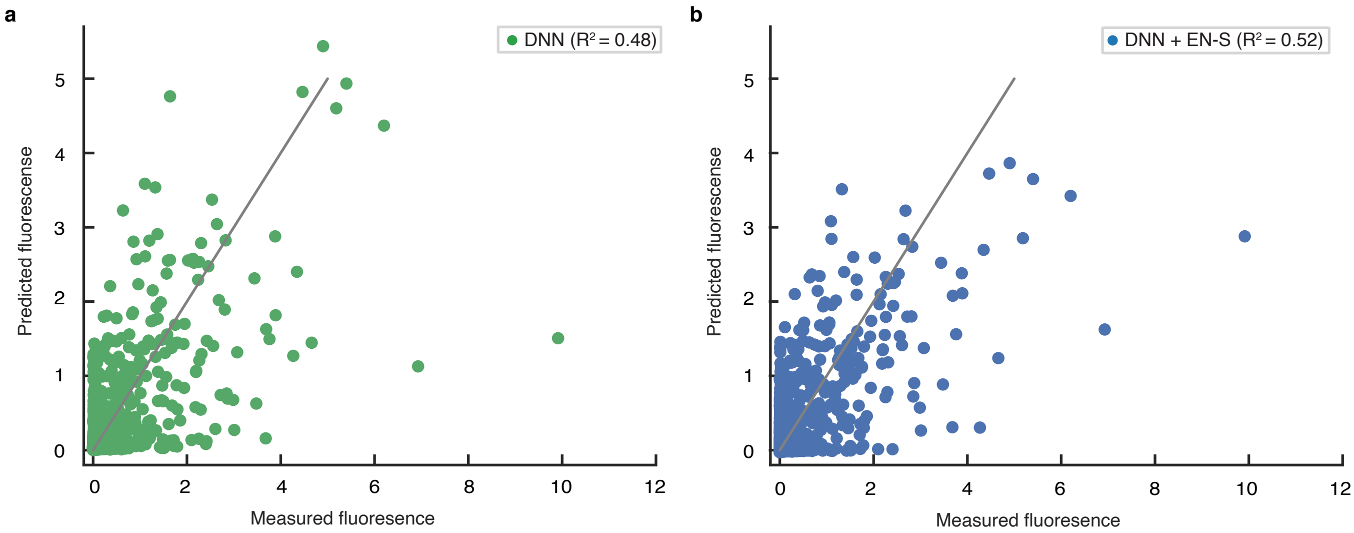

Figure S9: **The scatter plots of the measured and predicted protein functions. a**, DNN trained on the GB1 landscape. **b**, DNN with EN-S regularization trained on the GB1 landscape.

Supplementary References

[1] David Donoho. Compressed Sensing. *IEEE Transaction on Information Theory*, 52(4):1289–1306, 2006.

[2] Joel Tropp. Greed is good: Algorithmic results for sparse approximation. *IEEE Transactions on Information Theory*, 50(10):2231–2242, 2004.

[3] Robert Tibshirani. Regression Shrinkage and Selection via the Lasso. *Journal of the Royal Statistical Society: Series B (Methodological)*, 58(1):267–288, 1996.

[4] Xiao Li, Joseph Bradley, Sameer Pawar, and Kannan Ramchandran. SPRIGHT: A fast and robust framework for sparse Walsh-Hadamard transform. *arXiv preprint arXiv:1508.06336*, 2015.

[5] Tom Richardson and Rüdiger Urbanke. *Modern Coding Theory*. Cambridge University Press, 2008. Cambridge Books Online.

[6] Peter Elias. Error-free coding. *Transactions of the IRE Professional Group on Information Theory*, 4(4):29–37, Sep. 1954.

[7] Robin Scheibler, Saeid Haghighatshoar, and Martin Vetterli. A fast Hadamard transform for Signals with Sublinear Sparsity in the Transform Domain. *IEEE Transaction on Information Theory*, 61(4):2115–2132, 2015.

[8] Orhan Ocal, Swanand Kadhe, and Kannan Ramchandran. Low-degree Pseudo-Boolean Function Recovery Using Codes. In *2019 IEEE International Symposium on Information Theory (ISIT)*, pages 1207–1211. IEEE, 2019.

[9] Thomas Richardson and Rüdiger Urbanke. The capacity of low-density parity-check codes under message-passing decoding. *IEEE Transactions on Information Theory*, 47(2):599–618, 2001.

[10] Alex Tseng, Avanti Shrikumar, and Anshul Kundaje. Fourier-transform-based attribution priors improve the interpretability and stability of deep learning models for genomics. *Advances in Neural Information Processing Systems*, 33, 2020.

[11] Xiao Li, Joseph Bradley, Sameer Pawar, and Kannan Ramchandran. The SPRIGHT algorithm for robust sparse Hadamard transforms. In *2014 IEEE International Symposium on Information Theory*, pages 1857–1861. IEEE, 2014.

[12] Xiao Li and Kannan Ramchandran. An active learning framework using sparse-graph codes for sparse polynomials and graph sketching. In *Advances in Neural Information Processing Systems*, pages 2170–2178, 2015.

[13] Sameer Pawar and Kannan Ramchandran. FFAST: An algorithm for computing an exactly k-sparse DFT in $\mathcal{O}(k \log k)$ time. *IEEE Transactions on Information Theory*, 64(1):429–450, 2017.

[14] Mahdi Cheraghchi and Piotr Indyk. Nearly optimal deterministic algorithm for sparse Walsh-Hadamard transform. *ACM Transactions on Algorithms*, 13(3):1–36, 2017.

[15] Surojit Biswas, Grigory Khimulya, Ethan C Alley, Kevin M Esvelt, and George M Church. Low-N protein engineering with data-efficient deep learning. *Nature Methods*, 18(4):389–396, 2021.

[16] Aisha Khan, Duy Dinh, Dominique Schneider, Richard Lenski, and Tim Cooper. Negative epistasis between beneficial mutations in an evolving bacterial population. *Science*, 332(6034):1193–1196, 2011.

[17] J Arjan GM de Visser, Su-Chan Park, and Joachim Krug. Exploring the effect of sex on empirical fitness landscapes. *The American Naturalist*, 174(S1):S15–S30, 2009.

[18] Daniel Weinreich, Nigel Delaney, Mark DePristo, and Daniel Hartl. Darwinian evolution can follow only very few mutational paths to fitter proteins. *Science*, 312(5770):111–114, 2006.

[19] David Hall, Matthew Agan, and Sara Pope. Fitness epistasis among 6 biosynthetic loci in the budding yeast Saccharomyces cerevisiae. *Journal of Heredity*, 101(suppl_1):S75–S84, 2010.

[20] Frank Poelwijk, Michael Socolich, and Rama Ranganathan. Learning the pattern of epistasis linking genotype and phenotype in a protein. *Nature Communications*, 10(1):1–11, 2019.

[21] Nicholas C Wu, Lei Dai, C Anders Olson, James O Lloyd-Smith, and Ren Sun. Adaptation in protein fitness landscapes is facilitated by indirect paths. *eLife*, 5:e16965, 2016.

[22] Karen Sarkisyan, Dmitry Bolotin, Margarita Meer, Dinara Usmanova, Alexander Mishin, George Sharonov, Dmitry Ivankov, Nina Bozhanova, Mikhail Baranov, Onuralp Soylemez, et al. Local fitness landscape of the green fluorescent protein. *Nature*, 533(7603):397–401, 2016.

[23] J Arjan GM De Visser and Joachim Krug. Empirical fitness landscapes and the predictability of evolution. *Nature Reviews Genetics*, 15(7):480–490, 2014.

[24] Philip A Romero, Andreas Krause, and Frances H Arnold. Navigating the protein fitness landscape with gaussian processes. *Proceedings of the National Academy of Sciences*, 110(3):E193–E201, 2013.
