# Supplementary material for: Epistatic Net allows the sparse spectral regularization of deep neural networks for inferring fitness functions
Source: Nat Commun. 2021 Sep 1;12:5225. doi: 10.1038/s41467-021-25371-3 (PMC8410946; doi:10.1038/s41467-021-25371-3)
Supplement: Supplementary file 3 — Description of Additional Supplementary Files [file 41467_2021_25371_MOESM3_ESM.pdf]

Description of Additional Supplementary Files

File Name: Supplementary Data 1
Description: Prediction accuracies in four canonical bacterial datasets of Figure 2.

File Name: Supplementary Data 2
Description: Prediction accuracies in Entacmaea quadricolor datasets of Figure 3a.

File Name: Supplementary Data 3
Description: Epistatic error in Entacmaea quadricolor datasets of Figure 3b.

File Name: Supplementary Data 4
Description: Prediction accuracies in Entacmaea quadricolor datasets of Figure 3c.

File Name: Supplementary Data 5
Description: Prediction accuracies in avGFP and GB1 datasets of Figure 4.
