# Supplementary material for: Epistatic Net allows the sparse spectral regularization of deep neural networks for inferring fitness functions
Source: Nat Commun. 2021 Sep 1;12:5225. doi: 10.1038/s41467-021-25371-3 (PMC8410946; doi:10.1038/s41467-021-25371-3)
Supplement: Supplementary file 9 — Reporting Summary [file 41467_2021_25371_MOESM9_ESM.pdf]

Corresponding author(s): Kannan Ramchandran

Last updated by author(s): July 12, 2021

Reporting Summary

Nature Research wishes to improve the reproducibility of the work that we publish. This form provides structure for consistency and transparency in reporting. For further information on Nature Research policies, see our Editorial Policies and the Editorial Policy Checklist.

Statistics

For all statistical analyses, confirm that the following items are present in the figure legend, table legend, main text, or Methods section.

n/a	Confirmed	
☐	☒	The exact sample size (*n*) for each experimental group/condition, given as a discrete number and unit of measurement
☐	☒	A statement on whether measurements were taken from distinct samples or whether the same sample was measured repeatedly
☐	☒	The statistical test(s) used AND whether they are one- or two-sided<br>*Only common tests should be described solely by name; describe more complex techniques in the Methods section.*
☐	☒	A description of all covariates tested
☐	☒	A description of any assumptions or corrections, such as tests of normality and adjustment for multiple comparisons
☐	☒	A full description of the statistical parameters including central tendency (e.g. means) or other basic estimates (e.g. regression coefficient) AND variation (e.g. standard deviation) or associated estimates of uncertainty (e.g. confidence intervals)
☐	☒	For null hypothesis testing, the test statistic (e.g. *F*, *t*, *r*) with confidence intervals, effect sizes, degrees of freedom and *P* value noted<br>*Give P values as exact values whenever suitable.*
☒	☐	For Bayesian analysis, information on the choice of priors and Markov chain Monte Carlo settings
☒	☐	For hierarchical and complex designs, identification of the appropriate level for tests and full reporting of outcomes
☒	☐	Estimates of effect sizes (e.g. Cohen's *d*, Pearson's *r*), indicating how they were calculated

Our web collection on statistics for biologists contains articles on many of the points above.

Software and code

Policy information about availability of computer code

Data collection	We use data frame in Python to download and process the data.
Data analysis	We have developed a software in Python for EN regularization publicly available at https://github.com/amirmohan/epistatic-net. In this software we use standard ADAM optimizer for gradient descent. For the gradient boosting, random forest, and LASSO baselines we used standard packages from sklearn.

For manuscripts utilizing custom algorithms or software that are central to the research but not yet described in published literature, software must be made available to editors and reviewers. We strongly encourage code deposition in a community repository (e.g. GitHub). See the Nature Research guidelines for submitting code & software for further information.

Data

Policy information about availability of data

All manuscripts must include a data availability statement. This statement should provide the following information, where applicable:

- Accession codes, unique identifiers, or web links for publicly available datasets
- A list of figures that have associated raw data
- A description of any restrictions on data availability

The canonical bacterial fitness data used in Fig. 2 are publicly available in the github repository associated with the Ref. [18] (https://github.com/harmslab/notebooks-nonlinear-high-order-epistasis). The E. quadricolor fluorescent protein data used in Fig. 3 are publicly available in Supplementary Data 3 of Ref. 3 (https://doi.org/10.1038/s41467-019-12130-8). The avGFP protein data used in Fig. 4 are publicly available in the figshare data repository under accession code 3102154 (https://doi.org/10.6084/m9.figshare.3102154.v1). The GB1 protein data used in Fig. 4 are publicly available in Supplementary file 1 and Supplementary file 2 of Ref. 1 (https://elifesciences.org/articles/16965/figures). All other data generated or analyzed in this study are included in this published article (and in its accompanying Supplementary Information and Data). We have provided the links to access the data sets both in the manuscript and our github repository.

Field-specific reporting

Please select the one below that is the best fit for your research. If you are not sure, read the appropriate sections before making your selection.

- [x] Life sciences
- [ ] Behavioural & social sciences
- [ ] Ecological, evolutionary & environmental sciences

For a reference copy of the document with all sections, see nature.com/documents/nr-reporting-summary-flat.pdf

Life sciences study design

All studies must disclose on these points even when the disclosure is negative.

Sample size	The (sample) size of the data sets used are mentioned in the paper. We use all the experimental fitness data available to conduct our analysis. We demonstrate the performance of EN for a wide range of training sizes.
Data exclusions	No data was excluded in this work.
Replication	A software has been developed and released publicly for EN(-S) regularization algorithm at https://github.com/amirmohan/epistatic-net
Randomization	The collected data from the previously published articles were randomly split into test, train, and validation sets following standard procedure in statistical learning as described in the Methods section.
Blinding	Blinding is not typically used in the field. The standard procedure in machine learning is to keep a holdout unseen subset of data for validation.

Reporting for specific materials, systems and methods

We require information from authors about some types of materials, experimental systems and methods used in many studies. Here, indicate whether each material, system or method listed is relevant to your study. If you are not sure if a list item applies to your research, read the appropriate section before selecting a response.

Materials & experimental systems

n/a	Involved in the study	
x		Antibodies
x		Eukaryotic cell lines
x		Palaeontology and archaeology
x		Animals and other organisms
x		Human research participants
x		Clinical data
x		Dual use research of concern

Methods

n/a	Involved in the study	
x		ChIP-seq
x		Flow cytometry
x		MRI-based neuroimaging
